# Identification and Bioactivity of Compounds from the Mangrove Endophytic Fungus *Alternaria* sp.

**DOI:** 10.3390/md13074492

**Published:** 2015-07-20

**Authors:** Jinhua Wang, Weijia Ding, Ruimin Wang, Yipeng Du, Huanliang Liu, Xuehua Kong, Chunyuan Li

**Affiliations:** College of Materials and Energy (Formerly College of Science), South China Agricultural University, Guangzhou 510642, China; E-Mails: alanwongjh@sina.com (J.W.); dwjzsu@scau.edu.cn (W.D.); wangruimin47@sina.cn (R.W.); ohbilly@sina.com (Y.D.); LHLincredible@gmail.com (H.L.); akongxuehua@sina.com (X.K.)

**Keywords:** *Alternaria* sp., secondary metabolites, cyclopentenone, cyclohexenone, xanthone, antioxidant activity, antimicrobial activity

## Abstract

Racemic new cyclohexenone and cyclopentenone derivatives, (±)-(4R*,5S*,6S*)-3-amino-4,5,6-trihydroxy-2-methoxy-5-methyl-2-cyclohexen-1-one (**1**) and (±)-(4S*,5S*)-2,4,5-trihydroxy-3-methoxy-4-methoxycarbonyl-5-methyl-2-cyclopenten-1-one (**2**), and two new xanthone derivatives 4-chloro-1,5-dihydroxy-3-hydroxymethyl-6-methoxycarbonyl-xanthen-9-one (**3**) and 2,8-dimethoxy-1,6-dimethoxycarbonyl-xanthen-9-one (**4**), along with one known compound, fischexanthone (**5**), were isolated from the culture of the mangrove endophytic fungus *Alternaria* sp. R6. The structures of these compounds were elucidated by analysis of their MS (Mass), one and two dimensional NMR (nuclear magnetic resonance) spectroscopic data. Compounds **1** and **2** exhibited potent ABTS [2,2′-azino-bis(3-ethylbenzthiazoline-6-sulphonic acid)] scavenging activities with EC_50_ values of 8.19 ± 0.15 and 16.09 ± 0.01 μM, respectively. In comparison to Triadimefon, compounds **2** and **3** exhibited inhibitory activities against *Fusarium graminearum* with minimal inhibitory concentration (MIC) values of 215.52 and 107.14 μM, respectively, and compound **3** exhibited antifungal activity against C*alletotrichum musae* with MIC value of 214.29 μM.

## 1. Introduction

Marine microbes are recognized as an abundant source of biologically active natural products with structural diversity [1]. Among them, mangrove fungi constitute the second largest ecological group of marine fungi and produce a large number of chemicals with novel functions and structures [2]. *Alternaria* is a genus of ascomycete fungi with a widespread distribution, several species of which are known as major plant pathogens that may cause severe agricultural spoilage [3]. Chemical and bioactive investigations to *Alternaria* sp. from marine environments have led to several novel compounds with antimicrobial [4,5,6], cytotoxic [7,8,9], and MptpB (*Mycobacterium tuberculosis* protein tyrosine phosphatase B) inhibitory activities [10].

**Figure 1:**
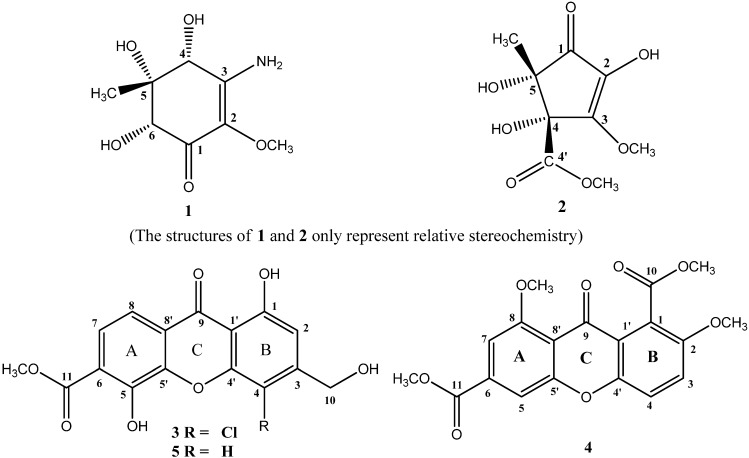
Structures of compounds **1**–**5**.

In our effort to search for bioactive metabolites from fungi derived from mangrove, we had screened extracts from a number of fungi. The methanol extract from the endophytic fungus, *Alternaria* sp. (collection No. R6), isolated from the root of *Myoporum bontioides* A. Gray, showed both antifungal and ABTS [2,2′-azino-bis(3-ethylbenzthiazoline-6-sulphonic acid)] radical scavenging activities. This prompted us to investigate the corresponding metabolites produced by the fungus. As a result, one new cyclohexenone derivative (**1**), one new cyclopentenone derivative (**2**), and two new xanthone derivatives (**3** and **4**) (Figure 1), together with a known compound, fischexanthone (**5**) [11] were isolated.

In previous investigations, a large number of xanthones had shown inhibitory activities against different fungi, such as *Fusarium oxysporum vasinfectum*, *Alternaria tenuis*, *Dreschlera oryzae* [12], *Aspergillus flavus* [13], *Gloeophyllum Candida krusei* and *Cryptococcus neoformans* [14]. While only a few monocyclic cyclohexenone and cyclopentenone derivatives had been reported to have antifungal activities. For example, (4*S*,5*S*,6*R*)-5,6-epoxy-4-hydroxy-3-methoxy-5-methylcyclohex-2-en-1-one and (4*R*,5*S*,6*R*)-4,5,6-trihydroxy-3-methoxy-5-methyl-cyclohex-2-en-1-one exhibited inhibitory activity against *Candida albicans* [15]. 5-(Hydroxyalkyl)-2-cyclopentenone derivatives, Hygrophorones A–G, showed fungicidal activities to *Cladosporium cucumerinum* [16]. Also, only several monocyclic cyclohexenone and cyclopentenone derivatives with hydroxyl substituents had been reported to possess antioxidant activities. For instance, 2-hydroxy-3-methyl-2-cyclopenten-1-one, exhibited great ABTS and moderate DPPH radical scavenging activities [17]. *cis*-2,4-Dihydroxy-2-(2-hydroxyethyl)cyclohex-5-en-1-one showed antioxidative effect on the auto-oxidation of linoleic acid [18]. In the study, details of the isolation, structure elucidation, and the results of antifungal and ABTS radical scavenging activities of the isolated compounds are reported herein.

## 2. Results and Discussion

### 2.1. Chemical Structure Elucidation

Compound **1** was isolated as colorless needle, whose molecular formula of C_8_H_13_O_5_N was determined by the positive HRESIMS (*m*/*z* 204.0870 [M + H]^+^, calcd. 204.0866). The IR spectrum indicated the presence of amino (3451, 3368 cm^−1^), hydroxyl (3242, 3191 cm^−1^), and carbonyl (1635 cm^−1^) groups. The ^1^H NMR spectrum of **1** (Table 1) indicated protons of one amino at δ_H_ 6.83 (s, 1H) and 6.30 (s, 1H), three hydroxyls at δ_H_ 5.94 (s, 1H, exchangeable), 4.94 (s, 1H, exchangeable) and 4.50 (s, 1H, exchangeable), two oxygenated methines at δ_H_ 4.22 (s, 1H) and 3.72 (s, 1H), one oxygenated methyl at δ_H_ 3.46 (s, 3H), and one methyl at δ_H_ 0.83 (s, 3H). The ^13^C NMR spectrum (Table 1) revealed the presence of one α,β-unsaturated ketone at δ_C_ 185.62, 156.36 and 126.93, one methoxyl at δ_C_ 58.13, one methyl at δ_C_ 13.39, two oxygenated methines at δ_C_ 76.99 and 71.55, and one quarternary carbon bearing an oxygen atom at δ_C_ 77.97. These data presumed compound **1** to be a cyclohexenone with one amino, three hydroxyls, one methoxyl, and one methyl substituents. The assignments of protons attached to their corresponding carbons were readily accomplished by the HSQC technique, which also confirmed the quaternary type of carbons at δ_C_ 185.62, 156.36, 126.93, and 77.97. In the HMBC spectrum, the correlations from the proton at δ_H_ 5.94(4-OH) to the carbon at δ_c_ 156.36 (C-3), from the proton at δ_H_ 3.46 (2-OCH_3_) to the carbon at δ_c_ 126.93 (C-2), and from the proton at δ_H_ 4.22 (H-4) to the carbons at δ_c_ 126.93 (C-2) and 156.36 (C-3), suggested that the –OH at δ_H_ 5.94 and the –OCH_3_ at δ_H_ 3.46 were respectively attached to C-4 at δ_c_ 71.55 and C-2 at δ_c_ 126.93. Simultaneously, the HMBC correlations from the proton at δ_H_ 4.22 (H-4) to the carbons at δ_c_ 13.39 (5-CH_3_) and 77.97 (C-5), and from the proton at δ_H_ 4.94 (5-OH) to the carbons at δ_c_ 71.55 (C-4), 77.97 (C-5) and 13.39 (5-CH_3_), indicated that the –OH at δ_H_ 4.50 and the methyl at δ_c_ 13.39 were both connected to the same quaternary carbon at δ_c_ 77.97 (C-5). Furthermore, the position of the –OH at δ_H_ 4.50 was determined to be at C-6 (δ_c_ 76.99) by the HMBC correlations from the proton at δ_H_ 4.50 (6-OH) to the carbons at δ_c_ 185.62 (C-1), 77.97 (C-5) and 76.99 (C-6), and from the proton at 3.72 (H-6) to the carbons at δ_c_ 185.62 (C-1), 71.55 (C-4), 77.97 (C-5), and 13.39 (5-CH_3_). Thus, the only position left to place the remaining –NH_2_ group of compound **1** was at C-3. Therefore, the planar structure of **1** was as shown in Figure 1. The relative configuration of the three hydroxyls in positions 4, 5, 6 was inferred as *cis*-relationship from the NOE correlations between 4-OH (δ_H_ 4.94) and 5-OH (δ_H_ 5.94), 4-OH and 6-OH (δ_H_ 4.50), and 5-OH and 6-OH, as well as no NOE correlations observed from 4-OH, 5-OH and 6-OH to 5-CH_3_ (δ_H_ 0.83) in the NOESY spectrum. So compound **1** was identified as (±)-(4R*,5S*,6S*)-3-amino-4,5,6-trihydroxy-2-methoxy-5-methyl-2-cyclohexen-1-one (4R*,5S*,6S* only represent the relative configuration). Compound **1** gave a zero specific rotation and showed a baseline CD (circular dichroism) curve (Figure S35), indicating a racemic mixture of the enantiomers [19], which results from the center of chirality (C-4, C-5, and C-6) of the cyclohexenone. This was confirmed also by chiral HPLC analysis, which afforded a doublet peak with cellulose-based stationary phase using *n*-hexane/isopropanol (*v*/*v* 90:10) as mobile phase (Figure S37). Based on the result of the analytical HPLC, they were attempted to separate by preparative chiral HPLC and using three types of chiral columns, but all were unsuccessful due to their small amounts.

**Table 1:** ^1^H and ^13^C NMR data of compounds **1** and **2**.

Position	1 ^a^		2 ^a^	
	δ_C_, mult.	δ_H_	δ_C_, mult.	δ_H_
1	185.62, C	-	197.95, C	-
2	126.93, C	-	132.96, C	-
3	156.36, C	-	157.68, C	-
4	71.55, CH	4.22 (s)	81.97, C	-
5	77.97, C	-	78.15, C	-
6	76.99, CH	3.72 (s)	-	-
2-OCH_3_	58.13, CH_3_	3.46 (s)	-	-
2-OH	-	-	-	9.00 (s)
3-NH_2_	-	6.30 (s), 6.83 (s)	-	-
3-OCH_3_	-	-	58.55, CH_3_	3.99 (s)
4-OH	-	5.94 (s)	-	6.10 (s)
5-CH_3_	13.39, CH_3_	0.83 (s)	22.16, CH_3_	1.16 (s)
5-OH	-	4.94 (s)	-	5.63 (s)
6-OH	-	4.50 (s)	-	-
4′	-	-	171.13, C	-
4′-OCH_3_	-	-	52.06, CH_3_	3.59 (s)

^a^ Measured in (CD_3_)_2_SO at 600 MHz (^1^H) and 150 MHz (^13^C).

Compound **2** was obtained as colorless needle, and its molecular formula was established as C_9_H_12_O_7_ (*m*/*z* 255.0471 [M + Na]^+^, calcd. 255.0475) on the basis of high-resolution ESIMS measurements, indicating that **2** contained 4 degrees of unsaturation. The IR spectrum indicated the presence of hydroxyl (3434, 3290 cm^−1^), ester (1746 cm^−1^), and carbonyl (1615 cm^−1^) groups. The ^1^H NMR spectrum of **2** (Table 1) showed the presence of one methyl at δ_H_ 1.16 (s, 3H), three hydroxyls at δ_H_ 9.00 (s, 1H, exchangeable), 6.10 (s, 1H, exchangeable) and 5.63 (s, 1H, exchangeable), and two methoxyls at δ_H_ 3.99 (s, 3H) and 3.59 (s, 3H). Among them, the methoxyl at δ_H_ 3.59 (s, 3H) was suggested to be the methoxycarbonyl group revealed by the HMBC correlations between 4′-OCH_3_ and C-4′ at δ_C_ 171.13. The ^13^C NMR spectrum (Table 1) showed 9 resolved signals including one α,β-unsaturated ketone at δ_C_ 197.95, 157.68 and 132.96, one ester carbonyl at δ_C_ 171.13, two methoxyls at δ_C_ 58.55 and 52.06, one methyl at δ_C_ 22.16, and two quaternary carbons at δ_C_ 81.97 and 78.15. It also showed that 3 of the 4 elements of unsaturation in **2** were due to the carbonyl and α,β-unsaturated ketone groups. The other one degree of unsaturation indicated that **2** contained a monocyclic ring. These NMR data suggested that compound **2** was a cyclopentenone derivative with one methyl, one methoxyl, one methoxycarbonyl, and three hydroxyls. Analysis of the HSQC spectrum provided the assignments of protons attached to their corresponding carbons, which also confirmed what carbons were belonging to the quaternary type. In the HMBC spectrum, the correlations from the hydroxyl proton at δ_H_ 9.00 (2-OH) to the carbons at δ_c_ 197.95 (C-1), 132.96 (C-2), and 157.68 (C-3), and from the methoxyl protons at δ_H_ 3.99 to the carbon at δ_c_ 157.68 (C-3), suggested that the –OH at δ_H_ 9.00 and the –OCH_3_ at δ_H_ 3.99 were respectively attached to C-2 at δ_c_ 132.96 and C-3 at δ_c_ 157.68. Simultaneously, the HMBC correlations from the hydroxyl proton at δ_H_ 6.10 (4-OH) to 157.68 (C-3) and 171.13 (C-4′), indicated that the -OH at δ_H_ 6.10 and the methoxycarbonyl (δ_c_ 171.13 and 52.06) were both connected to C-4 (δ_c_ 81.97). Furthermore, the HMBC correlations from the methyl protons at δ_H_ 1.16 (5-CH_3_) to the carbons at δ_c_ 197.95 (C-1), 81.97 (C-4) and 78.15 (C-5), and from the hydroxyl proton at δ_H_ 5.63 (5-OH) to the carbons at δ_c_ 197.95 (C-1), 81.97 (C-4), 78.15 (C-5) and 22.16 (5-CH_3_), indicated that the –OH at δ_H_ 5.63 and the methyl at δ_H_ 1.16 were both connected to C-5 (δ_c_ 78.15). Thus, the planar structure of **2** was elucidated as shown in Figure 1. The relative configuration of the hydroxyls in positions 4 and 5 was inferred as *cis*-relationship from the NOE correlations between 4-OH (δ_H_ 6.10) and 5-OH (δ_H_ 5.63) in the NOESY spectrum. Therefore, the structure of **2** was elucidated as (±)-(4S*,5S*)-2,4,5-trihydroxy-3-methoxy-4-methoxycarbonyl-5-methyl-2-cyclopenten-1-one (4S*,5S* only represent the relative configuration). No observable CD cotton effect (Figure S36) and optical rotation of **2** was detected, indicating that it was also a racemic mixture [19]. This was supported by subsequent HPLC analysis on a chiral cellulose-based phase collumn revealing a symmetrical doublet chromatographic peak by using *n*-hexane/isopropanol (*v*/*v* 95:5) as mobile phase (Figure S38). Unfortunately, resolution of the separation of **2** by preparative chiral HPLC was also unsuccessful.

More detailed experimental information for Chiral HPLC analysis of Compounds **1** and **2** are shown in Figures S37 and S38 at the Supplementary Information. Natural products are usually biosynthesized in one enantiomer form. Enantiomerically opposite products are less than 1% relative to the overall abundance of natural products, which often result from the action of stereochemically distinct enzymes that can give single and opposite enantiomeric products from achiral substrates [20]. For examples, (±)-tylopilusin A and (±)-tylopilusin B were two naturalfully substituted 2-cyclopenten-1-one racemates produced by one kind of microorganism, with similar chiral centers as compound **2**.

Compound **3** was isolated as yellow powder, and its molecular formula was determined as C_16_H_10_O_7_Cl based on the negative HRESIMS (*m/z* 349.0124 [M − H]^−^, calcd. 349.0121). The IR spectrum indicated the presence of hydroxyl (3169 cm^−1^), ester (1696 cm^−1^), carbonyl (1641 cm^−1^) and aromatic (1607 cm^−1^) groups. The ^1^H NMR spectrum (Table 2) exhibited the presence of a 1,2,3,4-tetrasubstituted benzene ring (ring A) at δ_H_ 7.49 (d, 9 Hz, 1H) and 7.68 (d, 9 Hz, 1H), and a 1,2,3,4,5-pentasubstituted benzene ring (ring B) at δ_H_ 6.98 (s, 1H), a hydroxyl at δ_H_ 12.20 (s, 1H), a hydroxymethyl at δ_H_ 4.65 (d, 5.4 Hz, 2H), and a methoxyl at δ_H_ 3.85 (s, 3H). The ^13^C NMR spectrum (Table 2) showed the presence of 16 carbon signals including one carbonyl at δ_C_ 180.73, one ester carbonyl at δ_C_ 167.09, one hydroxymethyl at δ_C_ 61.03, one methoxyl at δ_C_ 52.78, one tetrasubstituted benzene ring and one pentasubstituted benzene ring. These NMR data of **3** were closely comparable to those of the known compound **5**, fischexanthone [11]. The only obvious difference between **3** and **5** was ascribed to the substitution of a chlorine atom in ring B of **3**. This was proved by the HMBC and HRESIMS spectra. HMBC correlations from H-2 to C-1, 3, 4, 10 and 1′ indicated that the chlorine atom was attached to C-4. So, the structure of **3** was elucidated to be 4-chloro-1,5-dihydroxy-3-hydroxymethyl-6-methoxycarbonyl-xanthen-9-one, and was also named as 4-chlorofischexanthone.

**Table 2:** ^1^H and ^13^C NMR data of compounds **3** and **4**, *J* in Hz.

Position	3 ^a^		4 ^b^	
	δ_C_, mult.	δ_H_ (*J* in Hz)	δ_C_, mult.	δ_H_ (*J* in Hz)
1	159.55, C	-	120.71, C	-
2	108.59, CH	6.98 (s)	152.78, C	-
3	150.57, C	-	119.49, CH	7.52 (d, 9.6)
4	107.95, C	-	119.07, CH	7.38 (d, 8.4)
5	149.15, C	-	111.29, CH	7.72 (d, 1.2)
6	117.63, C	-	135.66, C	-
7	126.08, CH	7.49 (d, 9.0)	105.52, CH	7.41 (d, 1.2)
8	120.74, CH	7.68 (d, 9.0)	160.89, C	-
9	180.73, C	-	175.06, C	-
10	61.03, CH_2_	4.65 (d, 5.4)	167.74, C	-
11	167.09, C	-	165.52, C	-
1′	107.11, C	-	121.40, C	-
4′	150.78, C	-	149.18, C	-
5′	151.61, C	-	157.43, C	-
8′	117.57, C	-	114.18, C	-
1-OH	-	12.20 (s)	-	-
2-OCH_3_	-	-	56.96, CH_3_	3.92 (s)
5-OH	-	10.59 (s)	-	-
8-OCH_3_	-	-	56.79, CH_3_	4.06 (s)
10-OH	-	5.71 (s)	-	-
10-OCH_3_	-	-	53.03, CH_3_	4.09 (s)
11-OCH_3_	52.78, CH_3_	-	52.83, CH_3_	4.00 (s)

^a^ Measured in (CD_3_)_2_SO at 600 MHz (^1^H) and 150 MHz (^13^C); ^b^ Measured in CDCl_3_ at 600 MHz (^1^H) and 150 MHz (^13^C).

Compound **4** was obtained as white powder. Its molecular formula of C_19_H_16_O_8_ was determined by HRESIMS (*m/z* 395.0732 [M + Na]^+^, calcd. 395.0737). The IR spectrum of **4** indicated the presence of ester (1741, 1714 cm^−1^), carbonyl (1658 cm^−1^) and aromatic (1617, 1586 cm^−1^) groups. The ^1^H NMR spectral data (Table 2) showed the presence of a 1,3,4,5-tetrasubstituted benzene ring (ring A) at δ_H_ 7.41 (d, 1.2 Hz, 1H) and 7.72(d, 1.2 Hz, 1H), a 1,2,3,6-tetrasubstituted benzene ring (ring B) at 7.38 (d, 8.4 Hz, 1H) and 7.52 (d, 9.6 Hz, 1H), and four methoxyls at δ_H_ 4.09 (s, 3H), 4.00 (s, 3H), 3.92 (s, 3H) and 4.06 (s, 3H). The ^13^C NMR spectrum (Table 2) revealed the presence of 19 carbon signals including one carbonyl group at δ_C_ 175.06, two ester carbonyl groups at δ_C_ 165.52 and 167.74, four methoxyl groups at δ_C_ 52.83, 53.03, 56.79 and 56.96, and two tetrasubstituted benzene rings. These NMR data of **4** were similar to those of 6-carboxyl-2,8-dihydroxy-1-methoxycarbonyl-xanthen-9-one [21]. The obvious difference between **4** and 6-carboxyl-2,8-dihydroxy-1-methoxycarbonyl-xanthen-9-one was three methyl groups of the former replaced the three active hydrogen protons of the latter. This was proved by the HMBC spectrum, where correlations from δ_H_ 3.92 (s, 3H) to C-2 at δ_C_ 152.78, from δ_H_ 4.06 (s, 3H) to C-8 at δ_C_ 160.89, and from δ_H_ 4.00 (s, 3H) to C-10 at δ_C_ 167.74 were observed. The positions of all the four substituents were further confirmed to be the same as 6-carboxyl-2,8-dihydroxy-1-methoxycarbonyl-xanthen-9-one by analysis of the HMBC spectrum. Thus, the structure of **4** was elucidated as 2,8-dimethoxy-1,6-dimethoxycarbonyl-xanthen-9-one (Figure 1).

The structures, key HMBC and NOESY correlations of compounds **1**–**4** are shown in Figure 1 and Figure 2, respectively. More detailed spectra are available at the Supplementary Information (Figures S1–S34).

**Figure 2:**
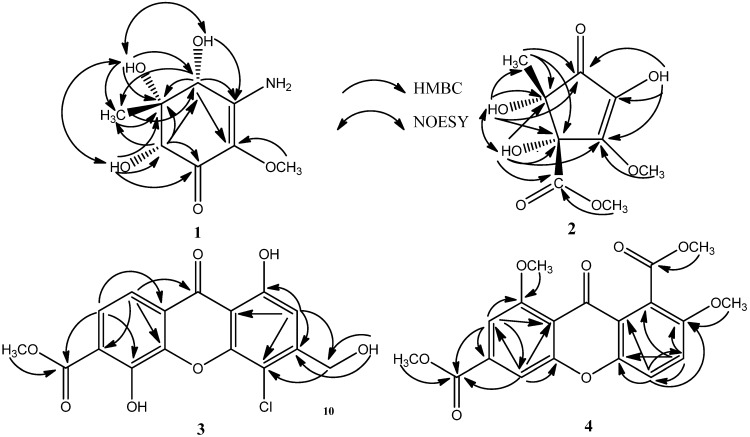
Key HMBC and NOESY correlations of compounds **1**–**4**.

### 2.2. Biological Activity

All of the new compounds were tested for ABTS radical scavenging activity. The results are showed in Table 3. Compounds **1** and **2** exhibited higher ABTS scavenging activities with EC_50_ values of 8.19 ± 0.15 and 16.09 ± 0.01 μM, respectively, than ascorbic acid (EC_50_ 17.14 ± 0.11 μM), which was used as the positive control, while compounds **3** and **4** did not exhibit antioxidant activities (EC_50_ > 500 μM) in this test. According to the data, compounds **1** and **2** both have marked ABTS radical scavenging activities. The ABTS radical scavenging activity curves of compounds **1** and **2** are available at the Supplementary Information (Figure S39). The antimicrobial activity of compounds **1**–**3** and **5** were evaluated *in vitro*. The results of their antibacterial and antifungal activities are summarized in Table 4. All the tested compounds exhibited antifungal activities against *Fusarium graminearum* with MIC values of 107.14–862.07 μM, in comparison to the positive control—Triadimefon (MIC 510.64 μM). Among them, the activities of compounds **2**, **3**, and **5** were higher than the positive control with MIC values of 215.52, 107.14 and 474.68 μM, respectively. Compound **3** also exhibited more potent antifungal activity against *Calletotrichum musae* with MIC value of 214.29 μM than Triadimefon (MIC 340.43 μM), while compounds **2** and **5** only showed moderate activities with MIC values of 862.07 and 474.68 μM, respectively. Compound **1** was inactive towards *Calletotrichum musae* (MIC > 1970.44 μM). Compound **3** bears a –Cl group at C-4 and exhibits greater antifungal activity against *Fusarium graminearum* and *Calletotrichum musae* than compound **5** with no substitute at C-4, suggesting that the –Cl group at C-4 is the key reason to increase the antifungal activity.

Additionally, compounds **1**, **2** and **5** displayed no activities neither against Gram positive bacterium *Staphyloccocus aureus* nor against Gram negative bacterium *Escherichia coli* with MIC value ≥1265.82 μM.

**Table 3:** ABTS radical scavenging activities of compounds **1**–**4** (EC_50_, μM) ^a^.

Compounds	1	2	3	4	Ascorbic Acid ^b^
EC_50_	8.19 ± 0.15	16.09 ± 0.01	>500.00	>500.00	17.14 ± 0.11

^a^ EC_50_ values are taken as means ± standard deviation from three independent experiment; ^b^ Used as a positive control.

**Table 4:** Antimicrobial activities of isolated compounds (minimal inhibitory concentration (MIC), μM).

	*Fusarium graminearum*	*Calletotrichum musae*	*Escherichia coil*	*Staphyloccocus aureus*
**1**	738.92	>1970.44	1970.44	1970.44
**2**	215.52	862.07	1724.14	1724.14
**3**	107.14	214.29	NT	NT
**5**	474.68	474.68	>1265.82	>1265.82
Cefradine ^a^	NT	NT	143.10	17.89
Kanamycin ^a^	NT	NT	1.54	1.54
Triadimefon ^a^	510.64	340.43	NT	NT

^a^ Used as a positive control. “NT” means “not detected”.

## 3. Experimental Section

### 3.1. General Experimental Procedures

IR spectra were obtained on a Nicolet 5DX-Fourier transform infrared spectrophotometer (Thermo Electron Corporation, Madison, WI, USA). All NMR experiments were recorded on a Bruker AVIII 600 MHz NMR spectrometer (Bruker BioSpin GmbH company, Rheinstetten, Germany), using deuterated dimethyl sulfoxide or chloroform as solvent and the residual solvent resonance as internal standard, and coupling constants (*J*) are in Hz. HR-ESI-MS were performed on LCMS-IT-TOF (Shimadzu, Janpan) mass spectrometer. Chromatography was carried out on a silica gel column (200–300 mesh; Qingdao haiyang chemicals Co., Ltd., Qingdao, China). CD data were measured on a Chirascan™ CD spectrometer (Applied Photophysics, London, UK). Optical rotations were measured on a Jasco P-1020 digital polarimeter. Chiral HPLC analyses were carried out using an Agilent 1100 liquid chromatograph equipped with a single pump, a UV-detector and a normal phase S-Chiral B cellulose-based column (Acchrom Technologies Co., Ltd., CHN, 5 μm, 150 × 4.6 mm) at a flow rate of 1.0 mL/min. All other reagents used were analytical grade.

### 3.2. Fungal Material and Fermentation

The strain of *Alternaria* sp. (collection No. R6) was isolated from the root of a marine semi-mangrove plant *Myoporum bontioides* A. Gray collected from the mangrove in Leizhou peninsula, Guangdong Province, China. Its species was identified by compared with the morphological characteristics with that of *Alternaria* sp. [9]. This fungus is stored in College of Materials and Energy (Formerly College of Science), South China Agricultural University, Guangzhou, China. The small agar slices bearing mycelia were cut from stock cultures and transferred aseptically to a 500 mL Erlenmeyer flask containing 250 mL of GYT medium (1% glucose, 0.1% yeast extract, 0.2% peptone, 0.2% crude sea salt), and incubated at 28 °C, 120 rpm for 6 days as seed culture. The fermentation was performed using 1 L Erlenmeyer flasks, each containing a rice medium (100 mL water, 100 g rice, 0.3 g crude sea salt). One milliliter of seed culture was added into each flask and incubated at room temperature for 30 days under static conditions.

### 3.3. Extraction and Isolation

The fermented rice substrate (75 flasks) was extracted repeatedly with methanol (4 × 100 mL for each flask) and solvent was combined and filtered through cheesecloth. The filtrate was concentrated to 5 L in vacuo below 50 °C and extracted five times using an equal volume of ethyl acetate and n-butyl alcohol in sequence, respectively. The combined ethyl acetate extract (48.6 g) was subjected to silica gel column chromatography and eluted with petroleum ether–ethyl acetate (90:10, 80:20, 70:30, 50:50, and 0:100) to afford Fractions 1–5 (1.9, 3.6, 6.2, 8.7, and 15.8 g, respectively), and the combined n-butyl alcohol extract (10.8 g) was subjected to silica gel column chromatography and eluted with ethyl acetate-methanol (100:0, 80:20) to afford Fractions 6–7 (2.8, 4.2 g, respectively) Fraction 4 (8.7 g) was subjected to silica gel column chromatography and eluted with petroleum ether-ethyl acetate in a gradient of ethyl acetate (petroleum ether-ethyl acetate, 80:20, 60:40, 50:50) to afford Fractions 4.1–4.3 (1.8, 1.7, and 2.5 g, respectively). Fractions 4.2, 4.3 and 7 were fractionated using Sephadex LH-20 column chromatography eluted with methanol-water to yield Fractions 4.2A–H (methanol-water 100:0, *v*/*v*), Fractions 4.3A–P (methanol-water 100:0, *v*/*v*) and Fractions 7A–K (methanol-water 67:33, *v*/*v*), respectively. Repeated slow recrystallization of fraction 7I (7 mg), 4.3F (6 mg), 4.3I (8 mg), 4.2D (12 mg) and 4.3M (8 mg) at room temperature from methanol-dichloromethane (60:40, *v*/*v*) yielded compound **1** (2.7 mg), compound **2** (2.5 mg), compound **3** (2.8 mg), compound **4** (3.4 mg) and compound **5** (4.5 mg), respectively.

(±)-(4R*,5S*,6S*)-3-Amino-4,5,6-trihydroxy-2-methoxy-5-methyl-2-cyclohexen-1-one (**1**): colorless needle; IR (KBr) ν_max_ 3451, 3368, 3242, 3191, 2980, 1635, 1040, 846 cm^−1^; ^1^H and ^13^C NMR data, see Table 1; ESIMS *m/z* 204 [M + H]^+^; HRESIMS *m*/*z* 204.0870 [M + H]^+^.

(±)-(4S*,5S*)-2,4,5-Trihydroxy-3-methoxy-4-methoxycarbonyl-5-methyl-2-cyclopenten-1-one (**2**): white needle; IR (KBr) ν_max_ 3434, 3290, 2962, 1746, 1708, 1615, 1261 cm^−1^; ^1^H and ^13^C NMR data, see Table 1; ESIMS *m/z* 255 [M + Na]^+^; HRESIMS *m*/*z* 255.0471 [M + Na]^+^.

4-Chloro-1,5-dihydroxy-3-hydroxymethyl-6-methoxycarbonyl-xanthen-9-one (**3**): yellow powder; IR (KBr) ν_max_ 3169, 2911, 1696, 1641, 1607, 1434, 1328, 824, 704 cm^−1^; ^1^H and ^13^C NMR data, see Table 2; ESIMS *m/z* 349 [M − H]^−^; HRESIMS *m*/*z* 349.0124 [M − H]^−^.

2,8-Dimethoxy-1,6-dimethoxycarbonyl-xanthen-9-one (**4**): white powder; IR (KBr) ν_max_ 2924, 2846, 1741, 1714, 1658, 1617, 1586, 1249, 1110, 798 cm^−1^; ^1^H and ^13^C NMR data, see Table 2; ESIMS *m*/*z* 395 [M + Na]^+^; HRESIMS *m*/*z* 395.0732 [M + Na]^+^.

### 3.4. The ABTS Radical Scavenging Activity Assay

The scavenging activity of ABTS radical was measured as described in previous literature [22] with minor modifications. The ABTS radical stock solution was preparred by reacting ABTS diammonium salt solution (7 mM) and potassium persulphate solution (2.45 mM) at room temperature in the dark for 12–16 h before use. The solution was diluted with ethanol until the absorbance at 734 nm reaching 0.7 ± 0.02 units. Three milliliters of diluted ABTS radical working solution was added to 1 mL of ethanolic solution of the test compound whose concentrations ranged from 3.13 to 200 μg/mL, and the final concentrations of the test compound in the mixed solution were 0.78–50 μg/mL in capped test tubes. The mixture was shaken vigorously and incubated at 30 °C in a water bath for 60 min in the dark. The absorbance was measured at 734 nm. Ascorbic acid was taken as a positive control. The scavenging ability was calculated as: ABTS radical scavenging activity (%) = [1 − (A1 − A2)/A0] × 100, where A_0_ is the absorbance in the lack of the test compound, A_1_ is the absorbance in the presence of the test compound and ABTS, and A_2_ is the absorbance in the lack of ABTS. The EC_50_ values (the concentration of the antioxidant required to scavenge 50% of ABTS present in the mixed solution) were obtained from liner regression analysis of the concentration-response curve plotted for each tested compound. In case the scavenging rate was found below 50% at the maximum tested concentration (50 μg/mL in the mixed solution), the test concentration would be directly increased to 500 μM to check whether the scavenging rate was still below 50%. If the scavenging rate was below 50% at 500 μM, it came to conclusion that EC_50_ was higher than 500 μM. Otherwise, further dilution would be made to acquire accurate value of the EC_50_.

### 3.5. Antimicrobial Activity Assay

Testing of antimicrobial activities of compounds was performed through determination of minimal inhibitory concentration (MIC) by the broth tube dilution method [23] in relation to fungi (*Fusarium graminearum*, *Calletotrichum musae*), and bacteria (*Escherichia coil*, *Staphyloccocus aureus*). Stock solution of compound was prepared in 5% (*v*/*v*) DMSO aqueous solution. Half a milliliter of the solution was further diluted with 0.5 mL of broth medium, and the final sample concentrations in the mixtures were 400, 200, 100, 50, 25, 12.5 and 6.25 μg/mL in capped test tubes. The tested microorganism taken from the culture medium was immediately diluted with sterilized water, and the final concentration of the microorganism was approximately 1 × 10^5^ c.f.u/mL in water. Ten microliters of microorganic suspension was added into each tube, and then bacteria were incubated at 37 °C for 24 h, fungi were incubated at 28 °C for 48 h. The MIC was defined as the lower concentration at which no microbial growth could be observed. As a positive control of growth inhibition, Cefradine and Kanamycin was used in the case of bacteria, Triadimefon in the case of fungi. Negative control of solvent influence was detected in parallel.

## 4. Conclusions

This investigation led to the isolation of one new cyclohexenone derivative, one new cyclopentenone derivative, and two new xanthone derivatives, which enriched the library of natural compounds. This was the first report showing that these compounds were produced by the fungus *Alternaria* sp. Compounds **1** and **2** exhibited higher ABTS radical scavenging activities with EC_50_ values of 8.19 ± 0.15 and 16.09 ± 0.01 μM, respectively, than ascorbic acid (EC_50_ 17.14 ± 0.11 μM), which revealed that they could be potential antioxidant drugs and/or lead compounds for constructing an antioxidant compound library. In comparison to Triadimefon, compounds **2** and **5** exhibited more potent inhibitory activity against *Fusarium graminearum*, and compound **3** exhibited more potent antifungal activity against both *Calletotrichum musae* and *Fusarium graminearum*, which suggested that the three compounds were worthy of consideration for the development and research of antifungal agents.

## Supplementary Files

Supplementary File 1
