# Supplementary material for: Identification and Bioactivity of Compounds from the Mangrove Endophytic Fungus Alternaria sp
Source: Mar Drugs. 2015 Jul 20;13(7):4492–504. doi: 10.3390/md13074492 (PMC4515629; doi:10.3390/md13074492)
Supplement: Supplementary File 1 [file marinedrugs-13-04492-s001.docx]

**Supplementary Information**

**Table of Contents**

**Figure S1.** ^1^H NMR spectrum (600 MHz) of compound **1** in (CD_3_)_2_SO.

**Figure S2.** Expansion of ^1^H NMR spectrum (600 MHz) of compound **1** in (CD_3_)_2_SO.

**Figure S3.** ^13^C NMR spectrum (150 MHz) of compound **1** in (CD_3_)_2_SO.

**Figure S4.** HSQC spectrum (600/150 MHz) of compound **1** in (CD_3_)_2_SO.

**Figure S5.** HMBC spectrum (600/150 MHz) of compound **1** in (CD_3_)_2_SO.

**Figure S6.** Expansion of HMBC spectrum (600/150 MHz) of compound **1** in (CD_3_)_2_SO.

**Figure S7.** NOESY spectrum (600 MHz) of compound **1** in (CD_3_)_2_SO.

**Figure S8.** HRESIMS spectrum of compound **1**.

**Figure S9.** IR spectrum of compound **1**.

**Figure S10.** ^1^H NMR spectrum (600 MHz) of compound **2** in (CD_3_)_2_SO.

**Figure S11.** Expansion of ^1^H NMR spectrum (600 MHz) of compound **2** in (CD_3_)_2_SO.

**Figure S12.** ^13^C NMR spectrum (150 MHz) of compound **2** in (CD_3_)_2_SO.

**Figure S13.** HSQC spectrum (600/150 MHz) of compound **2** in (CD_3_)_2_SO.

**Figure S14.** HMBC spectrum (600/150 MHz) of compound **2** in (CD_3_)_2_SO.

**Figure S15.** Expansion of HMBC spectrum (600/150 MHz) of compound **2** in (CD_3_)_2_SO.

**Figure S16.** NOESY spectrum of (600 MHz) of compound **2** in (CD_3_)_2_SO.

**Figure S17.** HRESIMS spectrum of compound **2**.

**Figure S18.** IR spectrum of compound **2**.

**Figure S19.** ^1^H NMR spectrum (600 MHz) of compound **3** in (CD_3_)_2_SO.

**Figure S20.** Expansion of ^1^H NMR spectrum (600 MHz) of compound **3** in (CD_3_)_2_SO.

**Figure S21.** ^13^C NMR spectrum (150 MHz) of compound **3** in (CD_3_)_2_SO.

**Figure S22.** HSQC spectrum (600/150 MHz) of compound **3** in (CD_3_)_2_SO.

**Figure S23.** HMBC spectrum (600/150 MHz) of compound **3** in (CD_3_)_2_SO.

**Figure S24.** Expansion of HMBC spectrum (600/150 MHz) of compound **3** in (CD_3_)_2_SO.

**Figure S25.** HRESIMS spectrum of compound **3**.

**Figure S26.** IR spectrum of compound **3**.

**Figure S27.** ^1^H NMR spectrum (600 MHz) of compound **4** in CDCl_3_.

**Figure S28.** Expansion of ^1^H NMR spectrum (600 MHz) of compound **4** in CDCl_3_.

**Figure S29.** ^13^C NMR spectrum (150 MHz) of compound **4** in CDCl_3_.

**Figure S30.** HSQC spectrum (600/150 MHz) of compound **4** in CDCl_3_.

**Figure S31.** HMBC spectrum (600/150 MHz) of compound **4** in CDCl_3_.

**Figure S32.** Expansion of HMBC spectrum (600/150 MHz) of compound **4** in CDCl_3_.

**Figure S33.** HRESIMS spectrum of compound **4**.

**Figure S34.** IR spectrum of compound **4**.

**Figure S35.** CD spectrum of compound **1**.

**Figure S36.** CD spectrum of compound **2**.

**Figure S37.** Chiral-HPLC profile of compound **1**.

**Figure S38.** Chiral-HPLC profile of compound **2**.

**Figure S39.** ABTS radical scavenging activity curves of compounds **1** and **2**.

**Figure S1.** ^1^H NMR spectrum (600 MHz) of compound **1** in (CD_3_)_2_SO.

**Figure S2.** Expansion of ^1^H NMR spectrum (600 MHz) of compound **1** in (CD_3_)_2_SO.

**Figure S3.** ^13^C NMR spectrum (150 MHz) of compound **1** in (CD_3_)_2_SO.

**Figure S4.** HSQC spectrum (600/150 MHz) of compound **1** in (CD_3_)_2_SO.

**Figure S5.** HMBC spectrum (600/150 MHz) of compound **1** in (CD_3_)_2_SO.

**Figure S6.** Expansion of HMBC spectrum (600/150 MHz) of compound **1** in (CD_3_)_2_SO.

**Figure S7.** NOESY spectrum (600 MHz) of compound **1** in (CD_3_)_2_SO.

**Figure S8.** HRESIMS spectrum of compound **1**.

**Figure S9.** IR spectrum of compound **1**.

**Figure S10.** ^1^H NMR spectrum (600 MHz) of compound **2** in (CD_3_)_2_SO.

**Figure S11.** Expansion of ^1^H NMR spectrum (600 MHz) of compound **2** in (CD_3_)_2_SO.

**Figure S12.** ^13^C NMR spectrum (150 MHz) of compound **2** in (CD_3_)_2_SO.

**Figure S13.** HSQC spectrum (600/150 MHz) of compound **2** in (CD_3_)_2_SO.

**Figure S14.** HMBC spectrum (600/150 MHz) of compound **2** in (CD_3_)_2_SO.

**Figure S15.** Expansion of HMBC spectrum (600/150 MHz) of compound **2** in (CD_3_)_2_SO.

**Figure S16.** NOESY spectrum (600 MHz) of compound **2** in (CD_3_)_2_SO.

**Figure S17.** HRESIMS spectrum of compound **2**.

**Figure S18.** IR spectrum of compound **2**.

**Figure S19.** ^1^H NMR spectrum (600 MHz) of compound **3** in (CD_3_)_2_SO.

**Figure S20.** Expansion of ^1^H NMR spectrum (600 MHz) of compound **3** in (CD_3_)_2_SO.

**Figure S21.** ^13^C NMR spectrum (150 MHz) of compound **3** in (CD_3_)_2_SO.

**Figure S22.** HSQC spectrum (600/150 MHz) of compound **3** in (CD_3_)_2_SO.

**Figure S23.** HMBC spectrum (600/150 MHz) of compound **3** in (CD_3_)_2_SO.

**Figure S24.** Expansion of HMBC spectrum (600/150 MHz) of compound **3** in (CD_3_)_2_SO.

**Figure S25.** HRESIMS spectrum of compound **3**.

**Figure S26.** IR spectrum of compound **3**.

**Figure S27.** ^1^H NMR spectrum (600 MHz) of compound **4** in CDCl_3_.

**Figure S28.** Expansion of ^1^H NMR spectrum (600 MHz) of compound **4** in CDCl_3_.

**Figure S29.** ^13^C NMR spectrum (150 MHz) of compound **4** in CDCl_3_.

**Figure S30.** HSQC spectrum (600/150 MHz) of compound **4** in CDCl_3_.

**Figure S31.** HMBC spectrum (600/150 MHz) of compound **4** in CDCl_3_.

**Figure S32.** Expansion of HMBC spectrum (600/150 MHz) of compound **4** in CDCl_3_.

**Figure S33.** HRESIMS spectrum of compound **4**.

**Figure S34.** IR spectrum of compound **4**.

**Figure S35.** CD spectrum of compound **1**.

**Figure S36.** CD spectrum of compound **2**.

**Figure S37.** Chiral-HPLC profile of compound **1**. Sample preparation: 0.5 mg solid in 1.5 mL isopropanol. Injection volume: 20 μL. Solvents: n-hexane: isopropanol = 90:10 (*v*:*v*). Chiral column: S-Chiral B cellulose-based column (Acchrom Technologies Co., Ltd.,CHN, 5 μm, 150 × 4.6 mm). Temperature: 25 °C. Flow rate: 1.0 mL/min. UV detection at λ = 254 nm.

**Figure S38.** Chiral-HPLC profile of compound **2**. Sample preparation: 0.5 mg solid in 1.5 mL isopropanol. Injection volume 20 μL. Solvents: n-hexane: isopropanol = 95:5 (*v*:*v*). Chiral column: S-Chiral B cellulose-based column (Acchrom Technologies Co., Ltd., CHN, 5 μm, 150 × 4.6 mm). Temperature: 25 °C. Flow rate: 1.0 mL/min. UV detection at λ = 254 nm.

**Figure S39.** ABTS radical scavenging activity curves of compounds **1** and **2**.

© 2015 by the authors; licensee MDPI, Basel, Switzerland. This article is an open access article distributed under the terms and conditions of the Creative Commons Attribution license (http://creativecommons.org/licenses/by/4.0/).
